# Supplementary material for: Cryo-EM structure of the mechanically activated ion channel OSCA1.2
Source: eLife. 2018 Nov 1;7:e41845. doi: 10.7554/eLife.41845 (PMC6235563; doi:10.7554/eLife.41845)
Supplement: Figure 1—source data 1. [file elife-41845-fig1-data1.docx]

**Figure 1-source data 1.** Data collection, processing, model refinement, and validation.

|  | OSCA1.2 in nanodiscs | OSCA1.2 in LMNG detergent |
| --- | --- | --- |
| **Data collection and processing** |  |  |
| Magnification | 36000 | 29000 |
| Voltage (kV) | 200 | 300 |
| Electron exposure (e–/Å^2^) | 60 | 60 |
| Defocus range (μm) | -0.4 to -2.2 | -1.0 to -2.6 |
| Pixel size (Å) | 1.15 | 1.03 |
| Symmetry imposed | C2 | C2 |
| Initial particle images (no.) | 675,536 | 326,398 |
| Final particle images (no.) | 76,797 | 134,337 |
| Map resolution (Å)  FSC threshold | 3.1  0.143 | 3.5  0.143 |
|  |  |  |
| **Refinement** |  |  |
| Map sharpening *B* factor (Å^2^) | -81 | -170 |
| Model composition  Protein residues  Ligands | 1268  0 | 1268  0 |
| R.m.s. deviations  Bond lengths (Å)  Bond angles (°) | 0.018  1.57 | 0.018  1.65 |
| Validation  MolProbity score  Clashscore  EMRinger score  Poor rotamers (%) | 1.31  2.89  4.45  0 | 1.04  1.3  2.73  0 |
| Ramachandran plot  Favored (%)  Allowed (%)  Disallowed (%) | 96.5  3.3  0.2 | 96.9  2.9  0.2 |
