# Supplementary material for: Cryo-EM structure of the mechanically activated ion channel OSCA1.2
Source: eLife. 2018 Nov 1;7:e41845. doi: 10.7554/eLife.41845 (PMC6235563; doi:10.7554/eLife.41845)
Supplement: Transparent reporting form [file elife-41845-transrepform.docx]

***eLife’s* transparent reporting form**

We encourage authors to provide detailed information *within their submission* to facilitate the interpretation and replication of experiments. Authors can upload supporting documentation to indicate the use of appropriate reporting guidelines for health-related research (see [EQUATOR Network](http://www.equator-network.org/%20)), life science research (see the [BioSharing Information Resource](https://biosharing.org/)), or the [ARRIVE guidelines](http://www.plosbiology.org/article/info:doi/10.1371/journal.pbio.1000412) for reporting work involving animal research. Where applicable, authors should refer to any relevant reporting standards documents in this form.

If you have any questions, please consult our Journal Policies and/or contact us: [editorial@elifesciences.org](mailto:editorial@elifesciences.org).

**Sample-size estimation**

- You should state whether an appropriate sample size was computed when the study was being designed
- You should state the statistical method of sample size computation and any required assumptions
- If no explicit power analysis was used, you should describe how you decided what sample (replicate) size (number) to use

Please outline where this information can be found within the submission (e.g., sections or figure legends), or explain why this information doesn’t apply to your submission:

For electrophysiological recordings, sample size indicates number of cells tested. Ns were determined based on previous publications from the lab and experience. A minimum of N=4 was the lowest number of cells included for each data set. Number of cells tested for each condition is mentioned in figure legends and in figure supplement data.

**Replicates**

- You should report how often each experiment was performed
- You should include a definition of biological versus technical replication
- The data obtained should be provided and sufficient information should be provided to indicate the number of independent biological and/or technical replicates
- If you encountered any outliers, you should describe how these were handled
- Criteria for exclusion/inclusion of data should be clearly stated
- High-throughput sequence data should be uploaded before submission, with a private link for reviewers provided (these are available from both GEO and ArrayExpress)

Please outline where this information can be found within the submission (e.g., sections or figure legends), or explain why this information doesn’t apply to your submission:

For electrophysiological recordings, each dataset is a compilation of at least two independent transfections, done on different days. The only time a cell was excluded was if the quality of the recording was poor and the leak current was very high. Necessary information is included in the materials and methods section.

**Statistical reporting**

- Statistical analysis methods should be described and justified
- Raw data should be presented in figures whenever informative to do so (typically when N per group is less than 10)
- For each experiment, you should identify the statistical tests used, exact values of N, definitions of center, methods of multiple test correction, and dispersion and precision measures (e.g., mean, median, SD, SEM, confidence intervals; and, for the major substantive results, a measure of effect size (e.g., Pearson's r, Cohen's d)
- Report exact p-values wherever possible alongside the summary statistics and 95% confidence intervals. These should be reported for all key questions and not only when the p-value is less than 0.05.

Please outline where this information can be found within the submission (e.g., sections or figure legends), or explain why this information doesn’t apply to your submission:

For EM maps, Fourier Shell Correlation (FSC) between independently refined half datasets is shown in Figure 1-figure supplement 1. An FSC calculation for map-to-model is shown in the same figure.

For electrophysiological recordings, statistical significance was computed using GraphPad Prism 6. Mann Whitney test was performed when comparison was made between 2 groups. Exact P values (alpha 0.05, two sided), test used, and sample size is indicated in figure legends. Mean value and SEM is also indicated in the figure legend.

(For large datasets, or papers with a very large number of statistical tests, you may upload a single table file with tests, Ns, etc., with reference to sections in the manuscript.)

**Group allocation**

- Indicate how samples were allocated into experimental groups (in the case of clinical studies, please specify allocation to treatment method); if randomization was used, please also state if restricted randomization was applied
- Indicate if masking was used during group allocation, data collection and/or data analysis

Please outline where this information can be found within the submission (e.g., sections or figure legends), or explain why this information doesn’t apply to your submission:

Samples were allocated into different groups based on the different isoforms tested for the study. Comparison was made between wildtype channel and mutant channel.

**Additional data files (“source data”)**

- We encourage you to upload relevant additional data files, such as numerical data that are represented as a graph in a figure, or as a summary table
- Where provided, these should be in the most useful format, and they can be uploaded as “Source data” files linked to a main figure or table
- Include model definition files including the full list of parameters used
- Include code used for data analysis (e.g., R, MatLab)
- Avoid stating that data files are “available upon request”

Please indicate the figures or tables for which source data files have been provided:

Cryo-EM maps of OSCA1.2 in nanodiscs and LMNG have been deposited to the Electron Microscopy Data Bank under accession codes XXXX and XXXX. Atomic coordinates of OSCA1.2 in nanodiscs and LMNG have been deposited to the PDB under IDs XXXX and XXXX.
